# Pink Cricket Balls May Be Visually Challenging at Sunset

**DOI:** 10.1177/2041669516687049

**Published:** 2017-02-01

**Authors:** Joshua M. Adie, Derek H. Arnold

**Affiliations:** School of Psychology and School of Human Movement and Nutrition Sciences, The University of Queensland, Brisbane, Australia; Department of Psychology, Northeastern University, Boston, MA, USA

**Keywords:** cricket, luminance contrast, colour contrast, speed perception

## Abstract

Cricket is one of the world’s most popular sports, followed by hundreds of millions of people. It can be dangerous, played with a hard ball flying at great velocities, and accidents have occasionally been fatal. Traditionally, cricket has been played during the day, using a dark red ball. Since the late 1970s, a shorter form of one-day cricket has been played both during the day and at night under floodlights. To overcome visibility issues, one-day cricket uses a white ball, and players wear coloured clothing. There is now a desire to play a traditional form of cricket during the day and at night, using a ‘pink’ ball while players wear white clothing. Concerns regarding visibility, and player and umpire safety, have been raised in this context. Here, we report that these concerns have a sound basis.

The desire to play a traditional form of cricket, during the day and at night under floodlights, creates a challenging visual environment. During play, the dominant light source undergoes dramatic changes, in intensity and composition. During traditional daytime playing hours, in optimal viewing conditions, the sun can be said to appear yellow due to the dominant wavelength of light it emits (Appleton, 1945). As the sun sets, sunlight must pass through an increasing volume of atmosphere to reach our eyes, intersecting increasing numbers of atmospheric particles as it does so. This disproportionately scatters higher frequency wavelengths of light, giving the sky a reddish hue due to the changed composition of light (McCartney, 1976). Once stadium floodlights become dominant, the composition of light at the playing surface shifts once more, back toward a more central wavelength within the visible spectrum.

Changes in the composition of light can alter the appearance of coloured objects, due to changes in the relative intensities of light reflected from different surfaces. The intensity of light reflected from a surface that looks red will, for instance, tend to become proportionally greater at sunset than at midday, relative to a surface that looks yellow or green. This reflectance ratio would change once more as the dominant light source shifts from being the setting sun to being the stadium floodlights (at night). When considering what type of impact these changes might have on player and umpire safety, one should be aware that human motion perception is driven predominantly by encoded differences in brightness. Hence, it is possible for an object to be visible, because it has a distinctive colour relative to its background, without people being able to accurately judge its speed, because it is nearly equally bright relative to the background (Cavanagh, Tyler, & Favreau, 1984; Lu, Lesmes, & Sperling,1999).

With these factors in mind, we turn to considering some measurements we took at a recent (October 25–28, 2016) first class day-night cricket match, played at the GABBA (a first-class ground) in Brisbane, Australia, using a recently developed pink cricket ball. We were able to take readings of the intensity of light reflecting off several surfaces on the second day/night of play. We were able to take some readings regularly during play, whereas others could only be taken intermittently, during breaks in play. We split readings between background surfaces, against which players and umpires need to see a ball, and the cricket balls themselves. Regularly sampled backgrounds included the sky, field (adjacent to the boundary rope), the *white* sightscreen and coloured stands. Regularly sampled balls included a traditional dark-red cricket ball, a white one-day cricket ball and a pink day-night cricket ball (all new, see Figure 2(a)). We were also able to sample the match ball and the pitch during breaks in play.

We have depicted luminance readings taken from regularly sampled balls and background surfaces in Figure 1, from 80 minutes before until 20 minutes after sunset (6:02 p.m.). Note that all red ball readings (we stopped red ball readings at 5:15 p.m., soon after the traditional time for stopping play in Brisbane) suggest that this ball is reliably *darker* than all pertinent backgrounds (the sky, field, stands and sightscreen). Similarly, white ball readings suggest it was reliably *brighter* than all pertinent backgrounds (bear in mind that it would not normally be seen against a white sightscreen, but a black one, further increasing its contrast). The pink ball, however, changes the polarity of its luminance contrast relative to pertinent backgrounds, including the sky, outfield and grand stands, during this period.

**Figure 1.**
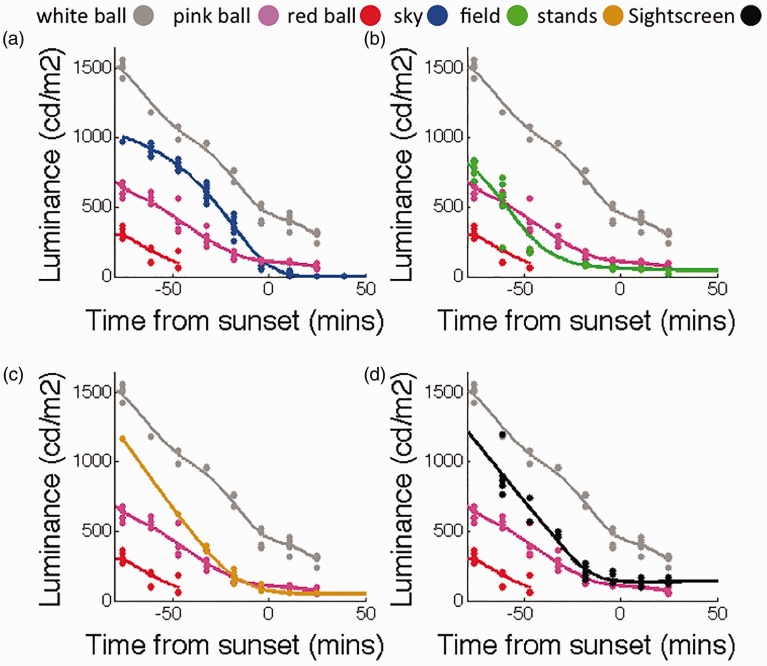
Luminance intensities (cd/m^2^) of recordings of reflectance’s from white (all panels, grey data), pink (all panels, pink), and red (all panels, red) cricket balls, the sky (a, blue), the outfield (b, green), grandstand (c, brown) and the sightscreen (d, black). All data are shown as a function of time from sunset (6:02 p.m.). Smoothed lines have been fit to all functions.

**Figure 2.**
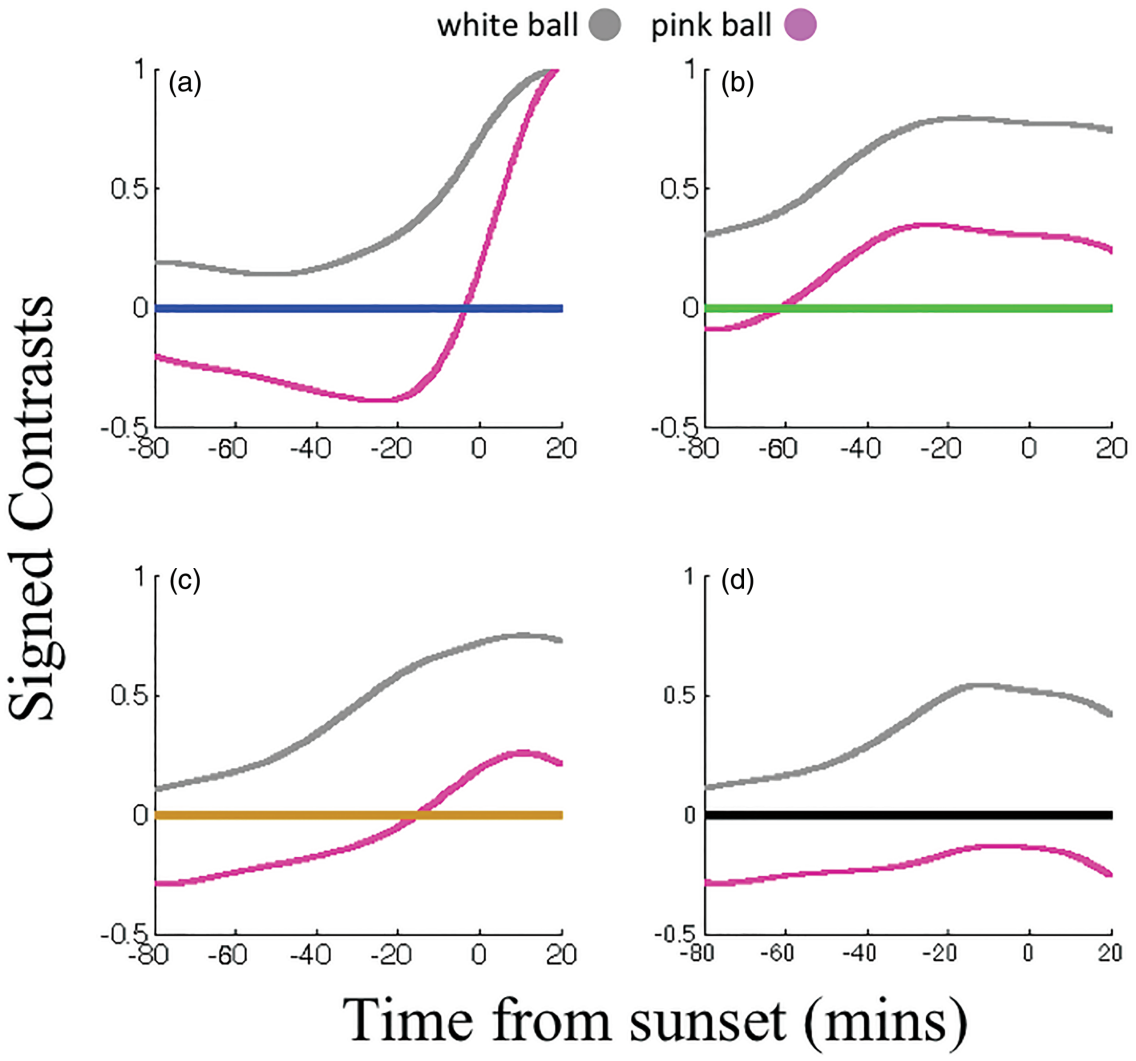
Signed Michelson contrast values between new white (grey functions) and new pink (pink functions) cricket balls, and pertinent backgrounds – the sky (a), field (b), stands (c) and white sightscreen (d). Contrasts are negatively signed when the ball was darker than the background, positive if it was brighter. Horizontal lines denote 0 contrast.

To highlight pink ball polarity reversals, in Figure 2, we have plotted signed Michelson contrast values, between smoothed pink and white ball luminance functions and those for pertinent backgrounds. These are negatively signed when the ball was darker than the background and positively signed when it was brighter. Luminance contrast polarity reversals are troubling, as a change in contrast polarity necessitates a period of transition, during which there will be minimal luminance contrast, and under such conditions, the human visual system can fail to accurately encode movement, with speed typically underestimated relative to higher contrasts (Stone & Thompson, 1992). Note that the times at which the pink ball changes polarity, relative to the sky and grandstands, are reasonably synchronized – about sunset, in this case during play.

Although we were able to sample the match ball and pitch less often, recordings suggest that these too were subject to a luminance contrast polarity reversal during play. In Figure 3(c), we show signed contrast values between the pink match ball, and a new pink ball, relative to the pitch in the middle of the playing area, at the end of the long break in play before sunset, and at the conclusion of play after sunset. These suggest that both these balls were brighter than the pitch ∼20 minutes before sunset, but darker than the pitch ∼150 minutes after sunset (when stadium lights were in full effect). These data show that a physical contrast polarity reversal has occurred. We do not know precisely when this happened, as we could not take recordings of the pitch and match ball during play. We believe this contrast polarity reversal was due to a change in the composition of light, with pink balls relatively better lit than the pitch by the sun near sunset, but worse by stadium floodlights after sunset.

**Figure 3.**
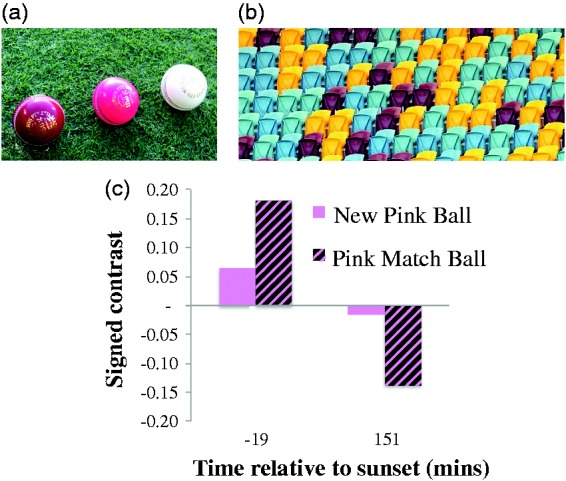
(a) Photo of the regularly sampled new cricket balls, against the field. (b) Photo of a section of grandstand. (c) Signed Michelson contrast values between the regularly sampled new pink cricket ball, and the pink match cricket ball, relative to the pitch. Negative contrasts indicate that the ball was darker than the pitch; positive values signify that the ball was brighter. Data are shown as a function of time from sunset (6:02 p.m.).

We have focused on the luminance contrasts of balls relative to pertinent backgrounds, as opposed to colour contrast. We suspect many people will be unaware that colour contrast contributes strongly to visibility but has little impact on speed perception (which is instead governed by luminance contrast). We will, however, mention one aspect of visibility. At dusk, when viewed against the grandstands during play, we found it entirely impossible to see a fast moving pink ball. This period coincided with a time at which the ball was nearly equal in average physical luminance relative to the stands, but in addition to this the stands were multi-coloured, and thus served as an effective mask for the fast moving pink ball. We suspect players and umpires would have experienced similar difficulty.

It remains to be seen how far our observations would generalize, and factors other than physical reflectances should be considered. For instance, light intensity reduces near sunset, so the composition of photoreceptors in the human eye used to encode input undergoes change, and this too can impact the appearance of coloured objects and speed perception (Barlow, 1957; Gegenfurtner, Mayser, & Sharpe, 2000). The relative intensities at which two surfaces look equally bright can also vary from physical equal luminance and differ for different individuals (Bilodeau & Faubert, 1997; Dobkins, Thiele, & Albright, 2000). Moreover, we tried to take recordings in a reliable fashion, sampling balls, for instance, from above, while avoiding specular highlights and stitching. Reflectances in natural viewing conditions will, however, vary greatly, depending on viewing angle and ball position, especially its elevation. Moreover, the balls used in matches now have dark stitching, possibly enhancing visibility. The reflectance of fields, pitches, and different match balls will also vary, and lighting conditions and dusk duration will change, depending on atmospheric conditions and geographic location.

With these caveats in mind, we do, however, believe our data speak to concerns that will generalize. First, the pink ball will inevitably undergo a change in contrast polarity relative to the sky. So, for a period, around dusk, players might experience difficulty in judging ball speed if they must see a pink ball moving with the sky as a backdrop. While it might not always undergo a reversal in contrast polarity relative to the field and pitch, we believe the change in the composition of light about sunset will inevitably lessen ball contrast against these surfaces at sunset. Behaviourally, this could result in the ball looking ‘blurred’ and in participants being slow to react. We believe these observations accord with concerns raised by players and umpires, and so encourage caution when considering changes to policies governing playing conditions.

In the interest of safety, it would be sensible to take reasonable precautions to ensure cricket is played in optimal visual conditions. Our data suggest visual conditions for a pink ball are degraded at sunset. This could be resolved by scheduling a sufficient break in play centred on sunset. Alternatively, a white-coloured ball sufficiently robust to withstand the rigours of test cricket might be developed. A third possibility is that glasses with appropriately coloured lenses could be used, to enhance the pink ball brightness relative to other surfaces.

## Appendix

Luminance recordings were taken via a ColorCAL MKII Colorimeter, interfaced with custom MATLAB software running on a Microsoft Surface Pro 4. Any readings exceeding 1100 cd/m^2^ were not analysed, due to concerns regarding the performance of the instrument at high light intensities, and because such readings might often relate to specular highlights, rather than to average surface reflectance.

Ball readings were taken from ∼20 cm above, at an angle, avoiding specular highlights. Sky readings were taken by pointing the Colorimeter directly up. Field and pitch recordings were taken from ∼20 cm above the ground at an angle of ∼45° to the playing surface. Sightscreen and grandstand recordings were taken by pointing the Colorimeter directly at the relevant surface, from a distance of ∼100 m.
